# FCGBP links hormonal imbalance and hepatic steatosis in PCOS–NAFLD comorbidity: an integrative bioinformatics and experimental study

**DOI:** 10.3389/fendo.2026.1750425

**Published:** 2026-05-18

**Authors:** Yi Yao, Min Si, Hongcheng Ding, Chengcheng Cao, Hua Yang, Qianshu Sun

**Affiliations:** 1 Department of Endocrine, Renmin Hospital, Hubei University of Medicine, Shiyan, Hubei, China; 2 Department of Emergency, Affiliated Dongfeng Hospital, Hubei University of Medicine, Shiyan, Hubei, China; 3 Department of Urology, Renmin Hospital, Hubei University of Medicine, Shiyan, Hubei, China

**Keywords:** FCGBP, hepatic steatosis, hormonal dysregulation, machine learning, nonalcoholic fatty liver disease, polycystic ovary syndrome

## Abstract

**Background:**

Polycystic ovary syndrome (PCOS) and nonalcoholic fatty liver disease (NAFLD) are prevalent endocrine-metabolic disorders with a high comorbidity rate. However, their shared molecular mechanisms remain largely unclear. This study aimed to identify and validate key co-expressed genes driving PCOS-NAFLD comorbidity.

**Methods:**

Transcriptomic datasets for PCOS (GSE34526) and NAFLD (GSE89632) were analyzed using differential expression combined with three machine learning algorithms (LASSO, Random Forest, and XGBoost). Robustness was assessed using leave-one-out (LOO) resampling and nested cross-validation. Functional enrichment and PPI network analyses identified FCGBP as a key shared gene. A DHEA + high-fat diet–induced rat model was used to simulate key features of PCOS–NAFLD, and DHT+FFA-treated KGN and HepG2 cells were used for *in vitro* validation, with FCGBP silencing and rescue experiments performed to assess its functional role.

**Results:**

FCGBP expression was significantly elevated in both ovarian and hepatic tissues in the rat model (P < 0.01). In KGN cells, FCGBP knockdown restored E2 secretion and upregulated CYP19A1 and HSD17B1 (P < 0.001). In HepG2 cells, FCGBP silencing reduced lipid accumulation and reversed SCD1 upregulation and ATGL downregulation (P < 0.05). These effects were partially reversed by FCGBP re-expression (rescue), further supporting the specificity of its regulatory role. These findings indicate that FCGBP is associated with both hormonal dysregulation and hepatic lipid metabolic abnormalities under hyperandrogenic conditions.

**Conclusion:**

This study identifies FCGBP as a potential molecular regulator in PCOS–NAFLD comorbidity and a candidate target for modulating both reproductive and metabolic dysfunctions.

## 1 Introduction

Polycystic ovary syndrome (PCOS) and nonalcoholic fatty liver disease (NAFLD) are two highly prevalent endocrine-metabolic disorders that significantly impair female reproductive function and hepatic health, respectively. The global prevalence of PCOS is estimated to range from 6% to 20% among women of reproductive age, whereas NAFLD affects more than 25% of the population and continues to increase worldwide (1–3). A growing body of clinical and epidemiological evidence has revealed a high comorbidity between PCOS and NAFLD, with some studies reporting a comorbidity rate of 40%–55%—markedly exceeding the prevalence observed in the general female population (4). Patients with coexisting PCOS and NAFLD exhibit greater risks for insulin resistance, type 2 diabetes mellitus, and cardiovascular diseases, along with poorer quality of life and long-term prognosis (5–7). Therefore, identifying shared molecular mechanisms and effective co-targets for intervention is of great clinical significance for improving precision therapy and reducing the burden of metabolic syndromes.

Although PCOS and NAFLD originate in different organ systems, they share several common pathophysiological features, including insulin resistance, chronic low-grade inflammation, dysregulated lipid metabolism, and hyperandrogenism (8, 9). Hyperandrogenism is not only a hallmark of PCOS but has also been implicated in the development and progression of NAFLD (10). Androgens can regulate hepatic lipid metabolism by modulating the expression of key enzymes involved in lipid synthesis and degradation, such as stearoyl-CoA desaturase 1 (SCD1) and adipose triglyceride lipase (ATGL) (11, 12). Simultaneously, elevated androgen levels impair estrogen biosynthesis in ovarian granulosa cells by downregulating aromatase (CYP19A1) and 17β-hydroxysteroid dehydrogenase 1 (HSD17B1) (13, 14). However, molecular mediators involved in the interaction between ovarian and hepatic dysfunction and simultaneously modulating reproductive and metabolic homeostasis remain largely unexplored. There is an urgent need to identify such biomarkers to deepen our understanding of PCOS–NAFLD comorbidity and develop dual-targeted therapeutic approaches.

With the advancement of multi-omics technologies and computational modeling, research into disease mechanisms is shifting from single-gene perspectives toward network-based and systems biology approaches. This transformation is particularly crucial in comorbidity studies, where traditional reductionist strategies often fail to capture inter-disease crosstalk and shared molecular drivers. In recent years, the integration of transcriptomic, epigenomic, and metabolomic data with machine learning algorithms has emerged as a powerful method for dissecting complex disease interactions (15). Algorithms such as Least Absolute Shrinkage and Selection Operator (LASSO) regression, Random Forest (RF), and eXtreme Gradient Boosting (XGBoost) enable feature selection, dimensionality reduction, and nonlinear modeling, making them well-suited for identifying key regulatory genes in high-dimensional biological data (16). Previous studies have demonstrated that machine learning algorithms, including LASSO, Random Forest, and gradient boosting methods, are effective tools for identifying key genes and improving predictive performance in complex diseases (17, 18). These approaches have been widely applied in cancer, autoimmune diseases, and cardiometabolic disorders, offering new opportunities for biomarker discovery and target prediction (19).

Based on this rationale, the present study aims to systematically identify co-expressed genes and regulatory networks involved in PCOS–NAFLD comorbidity using integrative transcriptomic analysis and machine learning–based screening. A candidate gene identified through this approach was further investigated in both *in vivo* and *in vitro* models to elucidate its potential role in androgen-induced reproductive and metabolic dysregulation. This study seeks to provide novel mechanistic insights and theoretical support for the development of dual-targeted interventions for PCOS and NAFLD.

## 2 Materials and methods

### 2.1 Data collection

Transcriptomic datasets related to NAFLD and PCOS were retrieved from the Gene Expression Omnibus (GEO) database (https://www.ncbi.nlm.nih.gov/geo/). The GSE89632 dataset was selected for NAFLD analysis, comprising 24 healthy liver tissue samples and 19 NAFLD samples. For PCOS analysis, the GSE34526 dataset was used, including 3 normal ovarian tissue samples and 7 PCOS samples. The diagnostic criteria for PCOS in this dataset were based on the original study from which the dataset was derived; however, detailed information regarding specific diagnostic standards (e.g., Rotterdam criteria) and clinical characteristics was not explicitly available in the GEO database. Since PCOS is a female-specific endocrine disorder, all samples in this dataset were derived from female subjects. It should be noted that the PCOS and NAFLD datasets were obtained from independent cohorts, and no patients with confirmed PCOS–NAFLD comorbidity were included in the public datasets. The raw gene expression data were downloaded and preprocessed using R software, including normalization and annotation based on the corresponding platform.

### 2.2 Differential expression analysis

After data normalization and quality control, differential gene expression analysis was performed separately for the NAFLD and PCOS datasets using the limma package in R. The empirical Bayes method was applied to shrink the variance estimates and improve statistical reliability, particularly given the limited sample size in the PCOS dataset. A linear model was constructed for each gene, and contrast matrices were defined to compare disease groups against healthy controls. Differentially expressed genes (DEGs) were identified based on the threshold of |log2 fold change (logFC)| ≥ 0.585 and adjusted p-value < 0.05. To further evaluate the reproducibility of DEG identification under small-sample conditions, leave-one-out (LOO) resampling and threshold sensitivity analyses were performed.

### 2.3 Identification of co-expressed genes and PPI network construction

To identify common molecular features between PCOS and NAFLD, the DEGs from both datasets were intersected to extract genes with consistent regulation trends (either upregulated or downregulated). These co-expressed genes were submitted to the STRING database (https://string-db.org/) to construct a protein–protein interaction (PPI) network. The analysis was performed using the “Multiple proteins” mode with a minimum interaction score of 0.4 (medium confidence). Only genes with confirmed or predicted interactions were retained for downstream visualization and network analysis.

### 2.4 Functional enrichment analysis

Gene Ontology (GO) and Kyoto Encyclopedia of Genes and Genomes (KEGG) enrichment analyses were conducted using the clusterProfiler package in R. GO terms included biological process (BP), molecular function (MF), and cellular component (CC). Enrichment significance was assessed by hypergeometric testing with a p-value < 0.05 and adjusted for multiple comparisons using the Benjamini–Hochberg method. Terms with false discovery rate (FDR) < 0.05 were considered statistically significant.

### 2.5 Machine learning–based gene selection

To identify key diagnostic genes among the co-expressed set, three machine learning algorithms were independently applied: LASSO, RF, and XGBoost.

For LASSO regression, the glmnet package in R was used. The gene expression matrix was input as the predictor variable (X), and sample classification (control vs. disease) as the response variable (Y). A 10-fold cross-validation was performed to determine the optimal regularization parameter (λ), and the λ value corresponding to the minimum mean cross-validated error (λ_min) was selected to balance model complexity and predictive performance, and genes with non-zero coefficients were retained.

For Random Forest, the randomForest package was used with parameters set to ntree = 1000, nPerm=500 and mtry = 4. The number of trees (ntree) was set to 1000 to ensure model stability and reduce variance, while mtry was determined based on empirical optimization to balance model accuracy and computational efficiency. Variable importance was assessed using the %IncMSE metric, and the top 10 ranked genes were selected.

XGBoost analysis was performed using the xgboost package with the following parameters: max_depth = 3, eta = 0.05, nrounds = 500, and objective = “binary:logistic”. These hyperparameters were selected based on commonly recommended settings and empirical tuning to prevent overfitting while maintaining model performance. Genes identified as important features by all three models were included for downstream validation.

### 2.6 Nested cross-validation and model evaluation

To evaluate model generalizability and reduce potential overfitting, nested cross-validation (Nested CV) was performed. In the outer loop, leave-one-out cross-validation (LOOCV) was applied, where one sample was used as the test set and the remaining samples were used for model training. In the inner loop, feature selection and hyperparameter tuning were conducted using cross-validation within the training set, without using information from the outer test set.

Model performance was evaluated using out-of-fold predictions to calculate the area under the receiver operating characteristic curve (AUC). To estimate uncertainty, bootstrap resampling was applied to compute 95% CI. In addition, the frequency of feature selection across nested cross-validation iterations was recorded to assess the stability of key genes.

### 2.7 Differential expression validation

Expression levels of candidate genes were extracted from both datasets, and the Wilcoxon rank-sum test was used to assess differences between disease and control groups. Analyses were performed in R, with *P* < 0.05 as the significance threshold.

### 2.8 Construction of nomogram and ROC curve analysis

A diagnostic nomogram model was constructed using the rms package in R, based on the expression levels of candidate genes and disease classification. Calibration curves were generated to evaluate model performance. The receiver operating characteristic (ROC) curve and area under the curve (AUC) were used to assess diagnostic accuracy.

### 2.9 Animal model and grouping

Female Sprague–Dawley (SD) rats (6–8 weeks old, 180–200 g; Institute of Laboratory Animal Sciences, Chinese Academy of Medical Sciences, Beijing, China) were housed under specific pathogen-free (SPF) conditions (temperature: 22 ± 2 °C; humidity: 50%–60%; 12 h light/dark cycle) with ad libitum access to food and water. All animal experiment procedures have been approved by the Experimental Animal Center of Hubei University of Medicine. The license number for the use of experimental animals is: SYXK (Guangdong) 2022-0002.

After one week of acclimatization, rats were randomly divided into two groups. Control rats were maintained on a standard chow diet and received daily subcutaneous injections of sesame oil (0.2 mL/rat). Model rats were fed a 60% high-fat diet and received daily subcutaneous injections of dehydroepiandrosterone (DHEA, 60 mg/kg/day; Sigma-Aldrich, USA, 53-43-0), dissolved in sesame oil (purity ≥98%, Aladdin, China), at a volume of 0.2 mL/rat for 21 consecutive days (20, 21). This model was used to mimic key metabolic and hormonal features associated with PCOS–NAFLD comorbidity, rather than to fully recapitulate all classical disease phenotypes. Body weight and general behavior were monitored daily. Once treatment ended, all rats were fasted overnight and euthanized with 5% isoflurane inhalation until deep anesthesia was achieved, as confirmed by loss of pedal withdrawal and corneal reflexes, followed by euthanasia by exsanguination under deep anesthesia for tissue collection. Death was confirmed by cessation of respiration and heartbeat, in accordance with the AVMA Guidelines for the Euthanasia of Animals.

### 2.10 Cell lines and experimental grouping

Human ovarian granulosa cell line KGN (SCSP-5495) and human hepatocellular carcinoma cell line HepG2 (TCHu 72) were obtained from the Cell Bank of the Chinese Academy of Sciences (Shanghai, China). KGN cells were cultured in Dulbecco’s Modified Eagle Medium/Nutrient Mixture F-12 (DMEM/F12), and HepG2 cells in high-glucose DMEM (both from Gibco, Thermo Fisher Scientific, USA, 11995065), supplemented with 10% fetal bovine serum (FBS, Gibco, USA, A5256701) and 1% penicillin-streptomycin (Solarbio, China, P1400). HepG2 cells were used as a widely adopted *in vitro* model for hepatic lipid metabolism studies, although their tumor-derived origin may not fully recapitulate normal hepatocyte physiology (22). All cells were maintained in a humidified incubator at 37 °C with 5% CO_2_.

After reaching 70%–80% confluence, cells were seeded into appropriate plates and divided as follows:

(1) KGN Cells: Hormonal Dysregulation Model: Control group, untreated; DHT+FFA group, treated with 30 μM dihydrotestosterone (DHT), 200 μM palmitic acid (PA), and 400 μM oleic acid (OA) (all from Sigma-Aldrich) for 24 hours (20); DHT+FFA+siNC group, transfected with negative control siRNA (siNC; GenePharma, China), then treated as above.; DHT+FFA+si-FCGBP group, transfected with siRNA targeting FCGBP (si-FCGBP; GenePharma), followed by the same DHT + FFA treatment.

(2) HepG2 Cells: Lipid Accumulation Model: Control group; DHT+FFA group, treated with 100 nM DHT, 200 μM PA, and 400 μM OA for 24 hours (23); DHT+FFA+siNC group, transfected with siNC, then treated as above; DHT+FFA+siNC+vector group, transfected with siNC siNC together with an empty vector, then treated as above; DHT+FFA+si-FCGBP group, transfected with si-FCGBP, followed by DHT + FFA treatment as above. DHT+FFA+si-FCGBP+FCGBP-rescue group, transfected with si-FCGBP and subsequently co-transfected with an FCGBP overexpression vector (siRNA-resistant construct), followed by DHT + FFA treatment as above.

### 2.11 Additional validation experiments

To minimize potential off-target effects, two independent siRNAs targeting FCGBP (si-FCGBP#1 and si-FCGBP#2) were used to validate knockdown efficiency in HepG2 cells.

For dose–response analysis, HepG2 cells were treated with increasing concentrations of DHT (0, 150, 300, 600, and 900 nM) in combination with FFA, and FCGBP expression was measured by qRT-PCR.

### 2.12 Quantitative real-time PCR

Total RNA was extracted from rat ovarian and liver tissues, as well as from KGN and HepG2 cells, using TRIzol reagent (Invitrogen, Thermo Fisher Scientific, USA) following the manufacturer’s protocol. RNA purity and concentration were measured with a NanoDrop 2000 spectrophotometer (Thermo Fisher Scientific, USA).

For gene expression analysis, qRT-PCR was performed using the UniPeak U+ One Step RT-qPCR SYBR Green Kit (Youjia Biotech, Guangzhou, China) in a 20 μL reaction system consisting of 10 μL 2× Master Mix, 0.4 μL each of forward and reverse primers (10 μM), 2 μL of cDNA template, and 7.2 μL RNase-free water. Reactions were run on a QuantStudio™ 5 Real-Time PCR System (Applied Biosystems, USA) under the following conditions: reverse transcription at 55 °C, initial denaturation at 95 °C for 30 s, followed by 40 cycles of 95 °C for 5 s and 60 °C for 30 s, ending with a melt curve analysis. Relative gene expression was calculated using the 2^^-^ΔΔCt^ method, with GAPDH as the internal control. Primer sequences are listed in **Table 1**.

**Table 1:** Primer sequences used for qRT-PCR.

Rat genes	Primer	Sequence(5′–3′)
FCGBP	FCGBP-F	GATACGCACAATACGCACATC
	FCGBP-R	CACACATCTGCTGGTATGTAGT
VAV3	VAV3-F	TAGGAACTACACTGAGCACC
	VAV3-R	CTACTGGTTTAGGTACACATGG
GAPDH	GAPDH-F	ACTCCCTCAAGATTGTCAGC
	GAPDH-R	AGTTGCTGTTGAAGTCACAGG
Human genes	Primer	Sequence (5′–3′)
FCGBP	FCGBP-F	CAATGCCTACAGTGAGTGCT
	FCGBP-R	TTGTGCGTATTGTGGTAGCAG
GAPDH	GAPDH-F	AAGATCATCAGCAATGCCTCC
	GAPDH-R	AGGTTTTTCTAGACGGCAGG

### 2.13 CCK-8 cell viability assay

KGN cells were seeded into 96-well plates (5 × 10³ cells/well) and subjected to the corresponding experimental treatments for 24 hours. After treatment, 10 μL of CCK-8 solution (Dojindo, Japan, CK04) was added to each well containing 100 μL of culture medium, and the plates were incubated at 37 °C for 2 hours. The optical density (OD) was measured at 450 nm using a BioTek Epoch microplate reader (Agilent Technologies, USA).

### 2.14 Biochemical detection of estrogen levels

After a 24-hour treatment, the culture supernatants from KGN cells were collected and centrifuged at 1,000 ×g for 10 minutes to remove debris. The levels of estradiol (E2) in the supernatants were measured using a commercial enzyme-linked immunosorbent assay (ELISA) kit (CUSABIO, Wuhan, China) according to the manufacturer’s instructions. All samples and standards were measured in duplicate. OD450 was measured using a BioTek Epoch microplate reader, and estrogen concentrations were calculated based on the standard curve.

### 2.15 Oil red O staining

After 24 hours of treatment, cells cultured in 6-well plates were washed twice with phosphate-buffered saline (PBS), fixed in 4% paraformaldehyde (Solarbio, China, P1110) for 20 minutes at room temperature, and stained with freshly prepared Oil Red O working solution (0.5% Oil Red O in isopropanol diluted 3:2 with distilled water; Sigma-Aldrich) for 30 minutes. After staining, cells were washed with PBS and counterstained with hematoxylin for nuclear visualization. Images were captured under an Leica DMi8 inverted microscope (Germany). For quantification, the dye was eluted with 100% isopropanol, and OD500 was measured using a BioTek Epoch microplate reader. Lipid content was expressed as relative absorbance normalized to the control group.

### 2.16 Western blot analysis

Total protein was extracted from KGN and HepG2 cells using RIPA lysis buffer (Beyotime, China, P0013B) with protease/phosphatase inhibitors. Protein concentrations were determined using a BCA Protein Assay Kit (Thermo Fisher, USA, 23227). Equal amounts of protein (30–50 μg per lane) were separated by 10% SDS-PAGE and blotted to 0.22 μm PVDF membranes (Millipore, USA, ISEQ00010). Membranes were blocked with 5% non-fat milk in TBST for 1 hour at room temperature and then incubated overnight at 4 °C with primary antibodies as follows: FCGBP (1:1,000, PC13168, Abmart, China), CYP19A1 (1:1,000, ab35604, Abcam, UK), HSD17B1 (1:1,000, ab51045, Abcam, UK), SCD1 (1:2,000, ab236868, Abcam, UK), ATGL (1:1,000, ab207799, Abcam, UK), and GAPDH (1:5,000, ab9485, Abcam, UK).

After washing, membranes were incubated with HRP-conjugated goat anti-rabbit IgG secondary antibody (1:5,000, ab205718, Abcam) for 1 hour at room temperature. Protein bands were visualized using an enhanced chemiluminescence (ECL) kit (Bio-Rad, USA) and imaged on a ChemiDoc™ MP Imaging System (Bio-Rad, USA). Band intensity was quantified using ImageJ software (NIH, USA), and expression levels were normalized to GAPDH.

### 2.17 Statistical analysis

All statistical analyses were conducted using SPSS 23.0 (IBM Corp., Armonk, NY, USA). Data are expressed as mean ± standard deviation (SD). For two-group comparisons, the independent-samples t-test was applied if the data conformed to normal distribution; otherwise, the Wilcoxon rank-sum test was used. Multi-group comparisons were performed using one-way ANOVA followed by Tukey’s *post hoc* test, or the Kruskal–Wallis test for non-normally distributed data. A two-sided P value < 0.05 was considered statistically significant. All experiments were independently repeated at least three times to ensure reproducibility.

## 3 Results

### 3.1 Identification of co-expressed differentially expressed genes in PCOS and NAFLD

**Figure 1:**
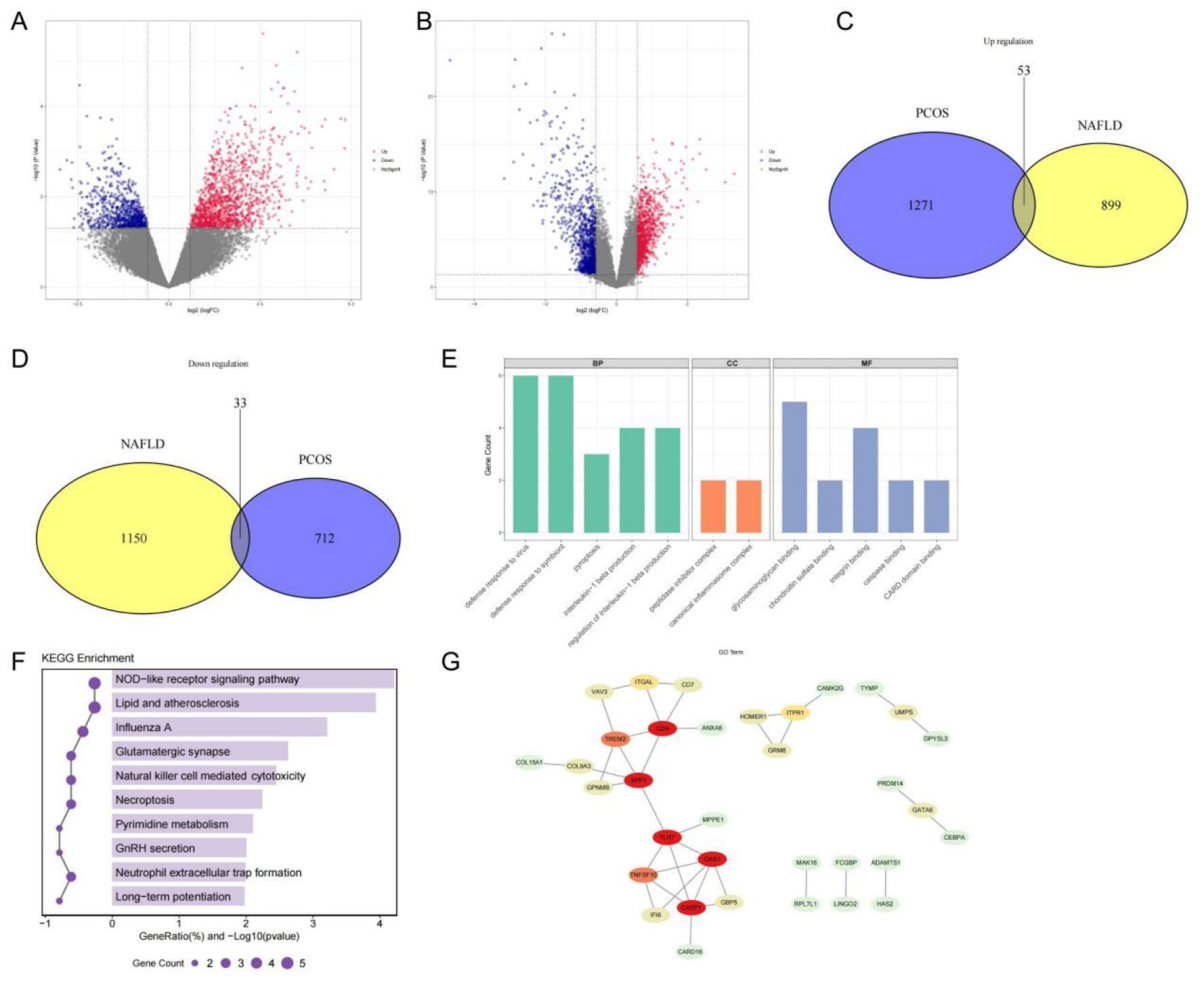
Identification and characterization of co-expressed DEGs between PCOS and NAFLD. **(A, B)** Volcano plots of DEGs in NAFLD and PCOS datasets. **(C, D)** Venn diagrams of co-upregulated and co-downregulated genes. **(E)** GO enrichment results across BP, CC, and MF categories. **(F)** KEGG pathway enrichment of co-expressed genes. **(G)** PPI network showing interaction hubs among shared DEGs.

To explore the molecular overlap between PCOS and NAFLD, we first performed transcriptome-level differential expression analyses using two transcriptomic datasets: GSE34526 (PCOS) and GSE89632 (NAFLD). Volcano plots revealed substantial numbers of DEGs in both datasets, with clear patterns of upregulation and downregulation relative to healthy controls (**Figures 1A, B**). By intersecting the DEG lists, we identified 53 genes consistently upregulated and 33 genes consistently downregulated across both diseases (**Figures 1C, D**). These 86 co-expressed DEGs were selected for subsequent functional analyses to elucidate shared pathogenic mechanisms.

To elucidate the biological relevance of the 86 co-expressed DEGs identified in both PCOS and NAFLD datasets, we performed GO and KEGG enrichment analyses.

GO analysis showed that these genes were enriched in immune-related biological processes such as defense response to virus, pyroptosis, and interleukin-1 beta production. Enriched cellular components included the canonical inflammasome complex and peptidase inhibitor complex, while molecular functions involved chondroitin sulfate binding, integrin binding, and caspase binding (**Figure 1E**). KEGG pathway analysis further revealed significant enrichment in signaling pathways such as NOD-like receptor signaling, lipid and atherosclerosis, GnRH secretion, and natural killer cell–mediated cytotoxicity, linking these genes to immune and endocrine-metabolic regulation (**Figure 1F**). In addition, a PPI network constructed via STRING and visualized in Cytoscape identified key hub genes (e.g., CASP1, TLR7, and OAS1), suggesting potential central regulators within the shared disease landscape (**Figure 1G**).

Together, these results highlight convergent immune-inflammatory and endocrine-metabolic processes underlying PCOS–NAFLD comorbidity and suggest potential molecular targets for further investigation.

To further evaluate the reproducibility of DEG identification under limited sample size, LOO resampling and threshold sensitivity analyses were performed. LOO analysis showed that key genes such as FCGBP and VAV3 were consistently identified across all iterations (reproducibility: 10/10), with relatively consistent effect sizes and rankings. In addition, sensitivity analysis using different logFC and FDR thresholds demonstrated that DEG detection was sensitive to multiple testing correction, with no genes retained under FDR < 0.05. These results indicate that, although the detected signals show good reproducibility, their statistical significance remains constrained by limited sample size. Detailed results are provided in **Supplementary Table 1**-**S3**.

### 3.2 Machine learning–based identification of hub genes shared in PCOS and NAFLD

Based on the co-expressed DEGs identified as described above and their assessed reproducibility, we employed three machine learning algorithms—LASSO regression, RF, and XGBoost—to screen the 86 co-expressed DEGs identified in the previous step.

**Figure 2:**
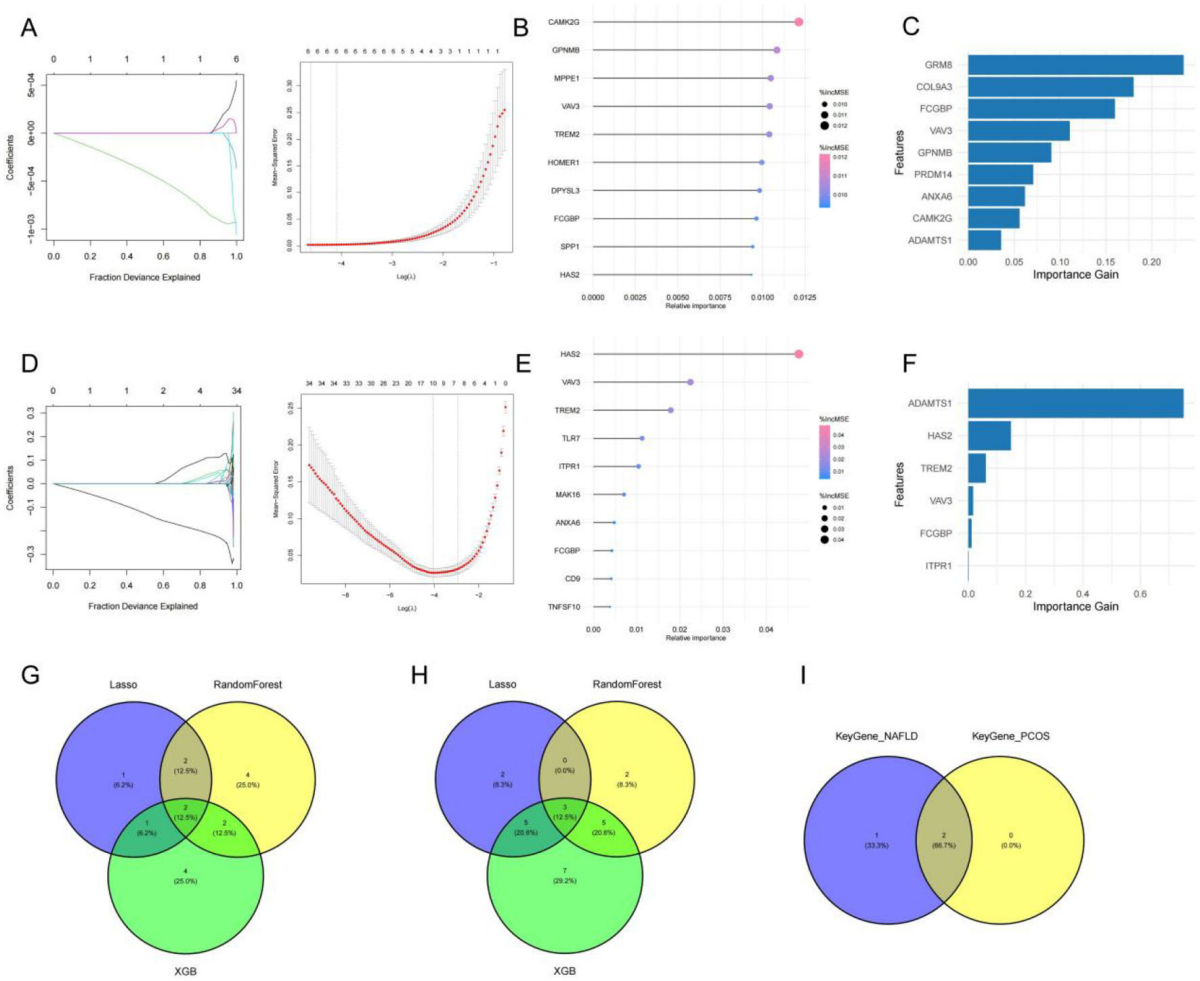
Machine learning–based identification of core genes shared in PCOS and NAFLD. **(A)** LASSO regression model for PCOS showing optimal lambda and nonzero coefficients. **(B, C)** Feature importance of top 10 genes in the PCOS dataset using Random Forest and XGBoost. **(D)** LASSO regression model for NAFLD dataset. **(E, F)** Feature importance of top genes in NAFLD via Random Forest and XGBoost. **(G, H)** Venn diagrams showing overlapping genes among the three models for PCOS and NAFLD. **(I)** Final intersection of key genes shared in both disease datasets, highlighting FCGBP and VAV3.

In the PCOS dataset, LASSO regression was performed using the glmnet package, with gene expression matrix as input (X) and disease status (control vs. PCOS) as output (Y). The model selected 6 genes with non-zero coefficients at the optimal lambda value (**Figure 2A**), In parallel, Random Forest (with mtry = 4) ranked gene importance based on %IncMSE, identifying the top 10 predictors including VAV3 and FCGBP (**Figure 2B**). XGBoost further prioritized features based on importance gain (**Figure 2C**). To further evaluate model generalizability and reduce potential overfitting, nested cross-validation was performed. The results showed that all models achieved relatively high out-of-fold AUC values, with LASSO exhibiting the best performance (AUC = 0.955), followed by Random Forest (AUC = 0.912) and XGBoost (AUC = 0.904). The ensemble model also demonstrated stable performance (AUC = 0.932), as summarized in **Supplementary Table 4**.

A similar modeling process was applied to the NAFLD dataset, where LASSO selected 34 nonzero genes (**Figure 2D**), and both RF and XGBoost produced consistent top-ranked candidates (**Figures 2E, F**). Cross-model comparison using Venn diagrams revealed FCGBP and VAV3 as the only two genes selected by all three algorithms in both datasets (**Figures 2G–I**). Further analysis based on nested cross-validation indicated that these genes were frequently selected across models, although selection frequencies varied (**Supplementary Table 5**).

Together, these findings highlight FCGBP and VAV3 as reproducibly identified candidate hub genes potentially mediating the shared pathogenesis of PCOS and NAFLD. Although both FCGBP and VAV3 were identified as candidate hub genes, FCGBP was prioritized for functional validation due to its stronger differential expression, higher ranking across models, and potential relevance to immune-metabolic regulation based on existing literature (24, 25).

### 3.3 FCGBP and VAV3 are significantly upregulated in PCOS and NAFLD and co-elevated in a DHEA + high-fat diet–induced rat model

**Figure 3:**
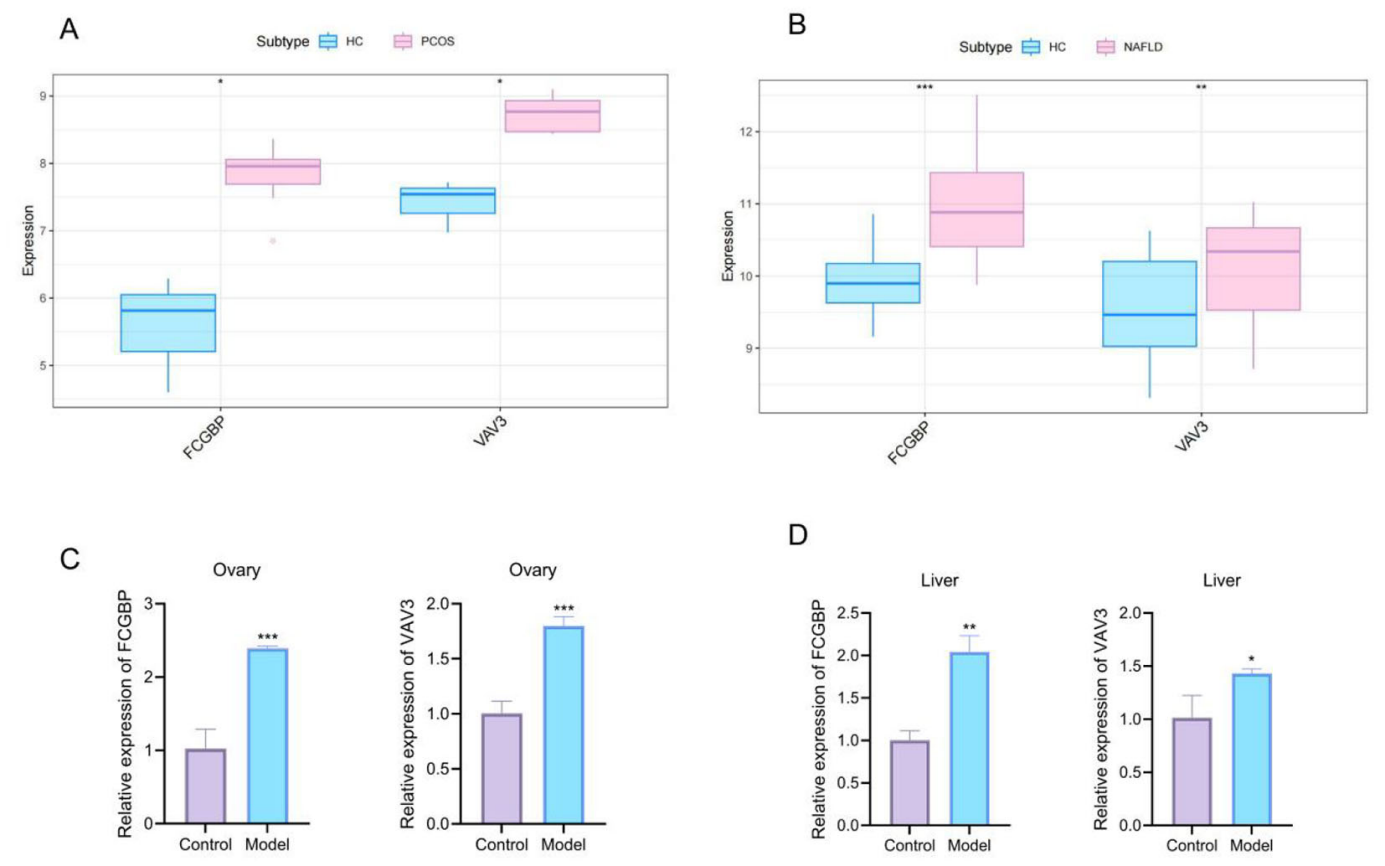
Expression validation of FCGBP and VAV3 in public datasets and comorbidity model. **(A, B)** FCGBP and VAV3 showed significant upregulation in PCOS and NAFLD patients in the GSE34526 and GSE89632 datasets, respectively. **(C, D)** qRT-PCR confirmed increased mRNA levels of FCGBP and VAV3 in ovarian **(C)** and hepatic **(D)** tissues of comorbidity model rats. *P < 0.05, **P < 0.01, ***P < 0.001 vs. Control.

To further validate the involvement of the previously identified hub genes in PCOS–NAFLD comorbidity, we examined the expression patterns of FCGBP and VAV3 in both public transcriptomic datasets and an *in vivo* rat model. In the GSE34526 dataset, both genes were significantly upregulated in ovarian tissues from PCOS patients compared to healthy controls (*P* < 0.05) (**Figure 3A**). Similarly, analysis of the GSE89632 dataset showed that FCGBP and VAV3 were significantly overexpressed in liver tissues from NAFLD patients (FCGBP: *P* < 0.001; VAV3: *P* < 0.01) (**Figure 3B**).

Next, we assessed the expression of these genes in ovarian and hepatic tissues of DHEA plus high-fat diet–induced PCOS–NAFLD comorbid rats. qRT-PCR analysis revealed that the mRNA levels of FCGBP and VAV3 were significantly elevated in the ovary of model rats compared to controls (*P* < 0.001) (**Figure 3C**). In the liver, the expression of FCGBP and VAV3 was also significantly upregulated (FCGBP: *P* < 0.01; VAV3: *P* < 0.05) (**Figure 3D**).

Together, these results confirm that FCGBP and VAV3 exhibit consistent upregulation in both human disease states and the comorbid rat model, suggesting their potential roles as shared regulators in the overlapping pathophysiology of PCOS and NAFLD.

### 3.4 Diagnostic potential of FCGBP and VAV3 for PCOS and NAFLD

**Figure 4:**
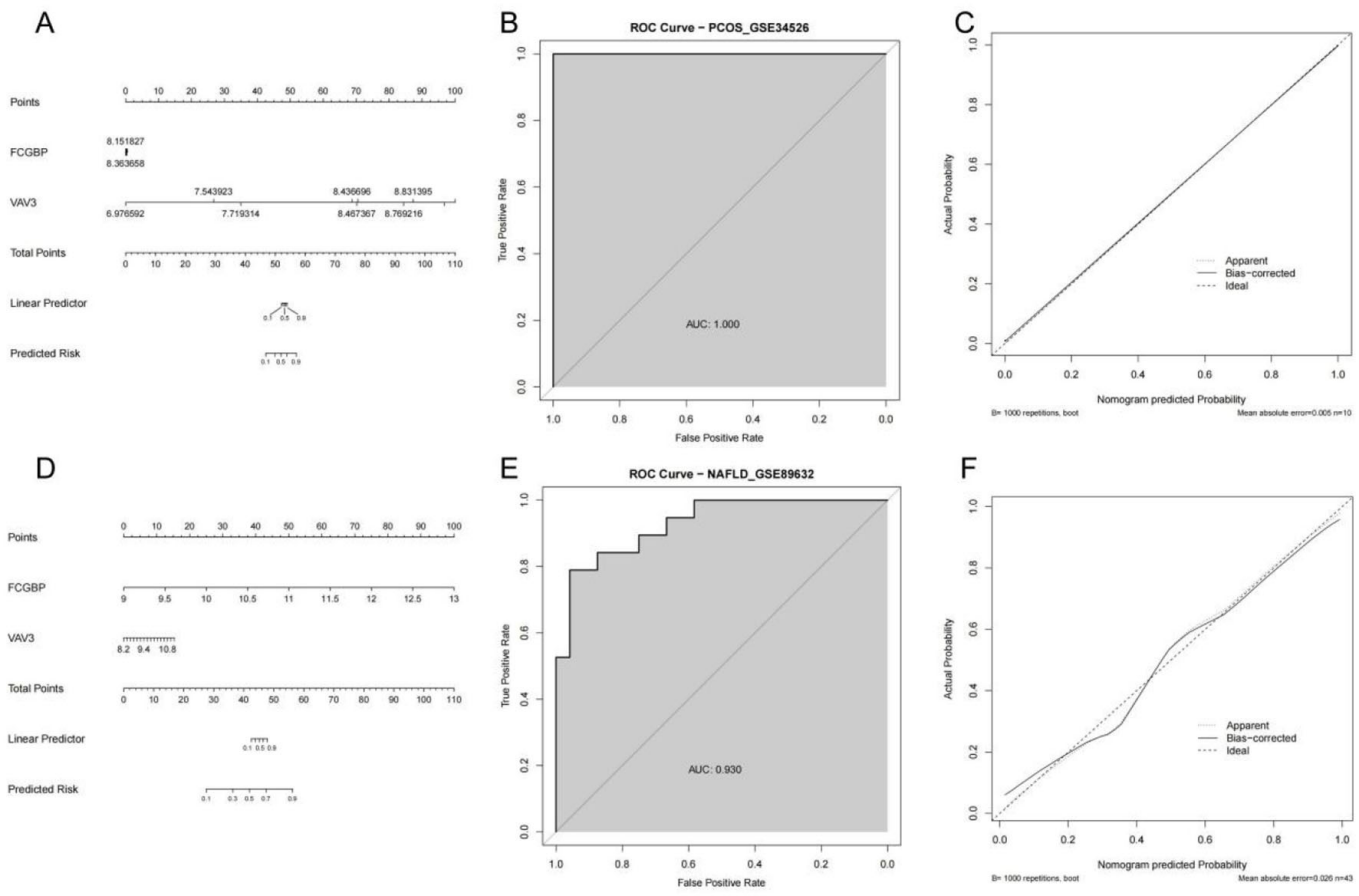
Diagnostic performance of FCGBP and VAV3 in PCOS and NAFLD. **(A, D)** Nomogram models for predicting PCOS and NAFLD risk using FCGBP and VAV3 expression levels. **(B, E)** ROC curves of the models in PCOS and NAFLD datasets. **(C, F)** Calibration curves showing good agreement between predicted and observed probabilities in both models.

To evaluate the diagnostic efficacy of the hub genes FCGBP and VAV3 in PCOS and NAFLD, we constructed disease-specific nomogram prediction models using logistic regression. In both datasets (PCOS and NAFLD), nomograms were developed based on the gene expression levels of FCGBP and VAV3 (**Figures 4A, D**).

The ROC analysis demonstrated that the combined gene model exhibited excellent discriminative power. Specifically, in the PCOS dataset, the area under the curve (AUC) reached 1.000, indicating perfect classification performance (**Figure 4B**). In the NAFLD dataset, the AUC was 0.930, also suggesting high diagnostic accuracy (**Figure 4E**).

Calibration curves were generated to assess the consistency between predicted and actual disease risk. Both models showed favorable calibration, as indicated by the bias-corrected curve closely aligning with the ideal diagonal line (**Figures 4C, F**).

Overall, these results suggest that FCGBP and VAV3 may serve as potential diagnostic biomarkers for PCOS and NAFLD, supporting their potential utility in clinical screening for comorbidity. Given that FCGBP showed a higher fold change, stronger statistical significance, and more consistent expression across both human and animal samples, it was selected for further functional validation.

### 3.5 FCGBP knockdown alleviates PCOS-like phenotypes in KGN cells

To investigate the functional role of FCGBP in PCOS, we established a DHT+FFA-induced PCOS-like model in KGN cells and silenced FCGBP using siRNA. To minimize potential off-target effects, two independent siRNAs targeting FCGBP were used and showed consistent knockdown efficiency (**Supplementary Figure 1**). In addition, FCGBP expression increased in a dose-dependent manner following DHT stimulation (**Supplementary Figure 2**), supporting the responsiveness of FCGBP to hyperandrogenic conditions.

**Figure 5:**
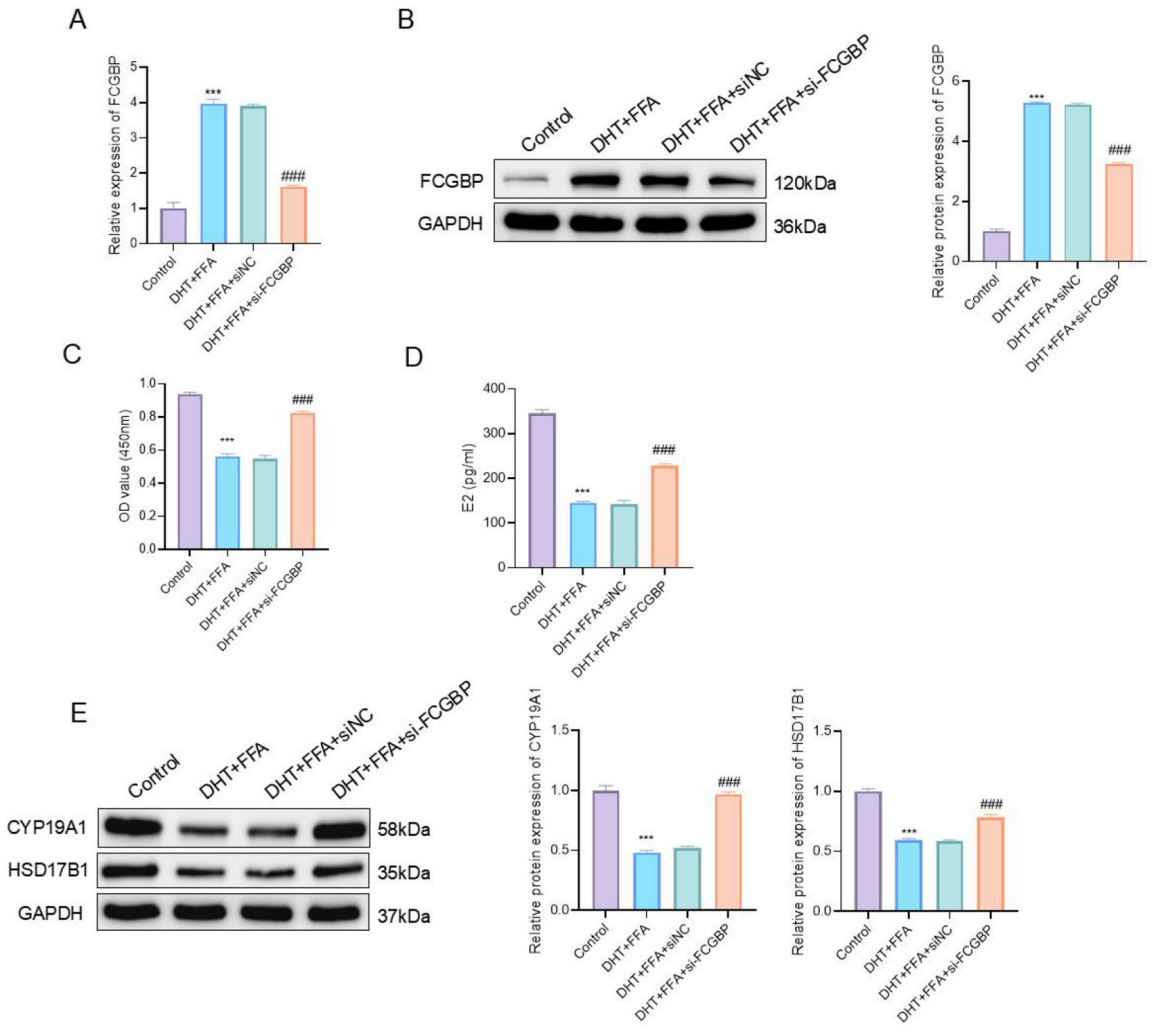
Functional validation of FCGBP in DHT+FFA-induced PCOS-like KGN cells. **(A)** Relative mRNA expression of FCGBP by qRT-PCR. **(B)** Protein expression of FCGBP analyzed by Western blot and corresponding quantification. **(C)** Cell viability measured by CCK-8 assay. **(D)** Estradiol (E2) levels determined by ELISA. **(E)** Protein expression of CYP19A1 and HSD17B1 analyzed by Western blot. Results are reported as mean ± SD (n = 3 independent experiments). ***P < 0.001 vs. Control; ###*P* < 0.001 vs. DHT+FFA+siNC.

qRT-PCR analysis showed that FCGBP expression was significantly elevated in model cells and was efficiently reduced by si-FCGBP transfection (*P* < 0.001, mean difference = 2.30, 95% CI: 2.02–2.57) (**Figure 5A**). Western blot analysis further confirmed that FCGBP protein expression was significantly increased in DHT+FFA-treated cells and effectively decreased following si-FCGBP transfection (P < 0.001, mean difference = 1.96, 95% CI: 1.82–2.10) (**Figure 5B**). CCK-8 assay revealed that FCGBP knockdown significantly restored cell viability compared to the model group (*P* < 0.001) (**Figure 5C**). ELISA results demonstrated that the E2 level, markedly reduced in the model group, was partially recovered upon FCGBP knockdown (*P* < 0.001, mean difference = 85.22, 95% CI: 68.30–102.10) (**Figure 5D**).

Western blot analysis further confirmed that protein levels of CYP19A1 and HSD17B1, which were downregulated in model cells, were significantly upregulated upon FCGBP knockdown (*P* < 0.001) (**Figure 5E**). These findings suggest that FCGBP may exert a detrimental role in PCOS-like cellular dysfunction, and its inhibition may alleviate related pathophysiological alterations.

### 3.6 FCGBP promotes lipogenesis and modulates lipid metabolism–related proteins

**Figure 6:**
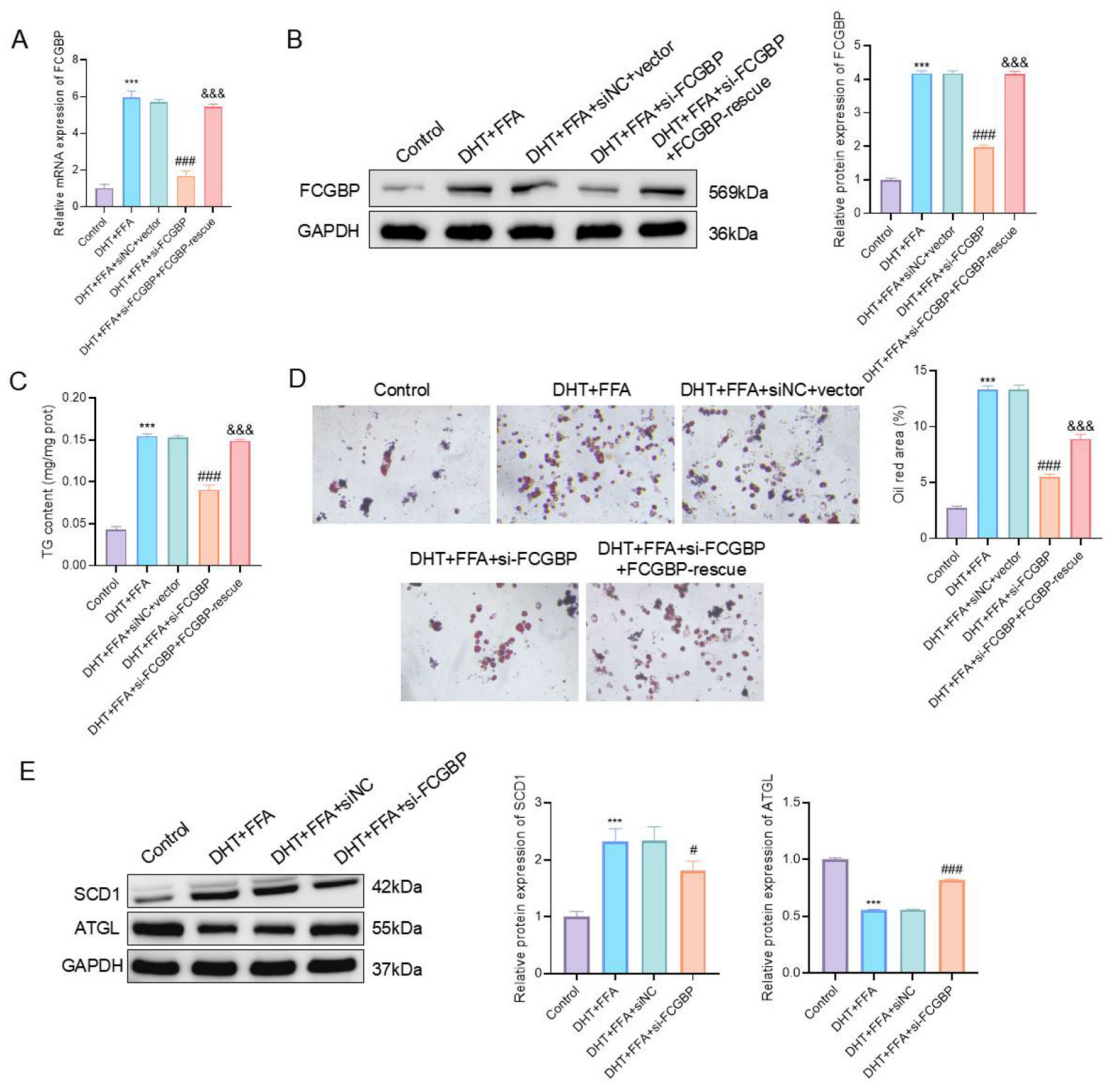
FCGBP is associated with increased lipid accumulation and regulates lipid metabolism–related protein expression in DHT+FFA-induced HepG2 cells. **(A)** qPCR analysis showing the relative mRNA expression of FCGBP. **(B)** Protein expression of FCGBP analyzed by Western blot and corresponding quantification. **(C)** Triglyceride (TG) content in HepG2 cells under different treatments. **(D)** Oil Red O staining and quantification of intracellular lipid accumulation. **(E)** Western blot analysis and quantification of lipogenic (SCD1) and lipolytic (ATGL) proteins. Results are reported as mean ± SD (n = 3 independent experiments). ***P < 0.001 vs. Control; #*P* < 0.05, ###*P* < 0.001 vs. DHT+FFA + siNC+vector or DHT+FFA + siNC. &&&*P* < 0.001 vs. DHT+FFA + si-FCGBP.

To further investigate the functional role of FCGBP in PCOS–NAFLD comorbidity, we established a DHT+FFA-induced HepG2 cell model. qPCR analysis revealed that FCGBP expression was significantly upregulated upon DHT+FFA treatment (*P* < 0.001, mean difference = 4.94, 95% CI: 5.57–4.31), while siRNA-mediated knockdown of FCGBP effectively reduced its expression (*P* < 0.001, mean difference = 3.99, 95% CI: 3.37–4.62). Importantly, re-expression of FCGBP partially restored its mRNA levels, confirming the efficiency and specificity of the intervention (**Figure 6A**). Western blot analysis further confirmed that FCGBP protein expression was significantly increased in DHT+FFA-treated cells and markedly decreased following si-FCGBP transfection (P < 0.001, mean difference = 2.21, 95% CI: 2.02–2.40). Consistently, FCGBP re-expression partially reversed the reduction in protein levels, further supporting the specificity of FCGBP modulation (**Figure 6B**). TG content analysis demonstrated a significant increase following DHT+FFA treatment, which was markedly reduced by FCGBP knockdown (P < 0.001, mean difference = 0.062, 95% CI: 0.053–0.071), while FCGBP rescue partially restored TG levels (P < 0.01, mean difference = 0.058, 95% CI: 0.0493–0.068), indicating a functional role of FCGBP in lipid accumulation (**Figure 6C**).

Oil Red O staining indicated a marked increase in intracellular lipid accumulation after DHT+FFA treatment, which was significantly attenuated by FCGBP silencing (*P* < 0.01) Notably, FCGBP re-expression partially reversed this effect, further confirming the involvement of FCGBP in lipid accumulation (**Figure 6D**). At the protein level, Western blotting demonstrated that DHT+FFA increased the expression of lipogenic marker SCD1 (*P* < 0.001, mean difference = 1.33, 95% CI: 0.83–1.82), and decreased the lipolytic protein ATGL (*P* < 0.001, mean difference = 0.45, 95% CI: 0.42–0.47), while FCGBP knockdown reversed these effects (SCD1: *P* < 0.05, mean difference = 0.52, 95% CI: 0.029–1.02; ATGL: *P* < 0.001, mean difference = 0.26, 95% CI: 0.23–0.29) (**Figure 6E**).

These results suggest that FCGBP is associated with increased lipid accumulation and may contribute to the expression of lipid metabolism–related proteins, potentially contributing to the hepatic steatosis observed in PCOS–NAFLD comorbidity.

## 4 Discussion

In this study, we systematically identified co-expressed DEGs associated with the comorbidity of PCOS and NAFLD through integrative transcriptomic analysis combined with multiple machine learning algorithms. Among these genes, FCGBP emerged as a candidate key gene and was functionally validated in both *in vivo* and *in vitro* models. To our knowledge, FCGBP may represent a candidate mediator associated with both ovarian hormonal imbalance and hepatic lipid dysregulation under hyperandrogenic conditions. These findings provide new molecular insights into PCOS–NAFLD comorbidity and offer a theoretical basis for dual-targeted therapeutic strategies.

FCGBP (Fc fragment of IgG binding protein) is a high-molecular-weight mucin-like glycoprotein initially identified in intestinal epithelial cells. It is widely expressed in barrier tissues such as the gastrointestinal tract, airways, and reproductive tract, and is known for its roles in IgG binding, epithelial adhesion, and maintenance of mucosal integrity (26). Previous studies have mostly associated FCGBP with mucosal immunity, chronic inflammation, and tumor microenvironment modulation, particularly in diseases such as inflammatory bowel disease, pulmonary fibrosis, and gastric cancer (27). However, its role in metabolic or endocrine disorders remains largely unexplored, and its involvement in the intersection of hormonal, metabolic, and immune networks has not been previously reported. Structurally, FCGBP contains multiple cysteine-rich domains, suggesting potential for complex protein–protein interactions (28). In this study, FCGBP was identified as one of the top-ranked co-expressed DEGs in both PCOS and NAFLD datasets and frequently selected by multiple machine learning algorithms, positioning it as a potential regulator at the intersection of reproductive and metabolic dysfunction.

Functional experiments further confirmed the dual regulatory role of FCGBP under hyperandrogenic stress. In DHT+FFA-induced KGN cells, FCGBP expression was significantly upregulated, and its knockdown effectively restored the expression of key steroidogenic enzymes, CYP19A1 and HSD17B1, while elevating E2 secretion. In hepatocyte models, silencing FCGBP reversed the upregulation of the lipogenic marker SCD1 and rescued ATGL expression, resulting in reduced lipid accumulation. These findings suggest that FCGBP is associated with dysfunction in both ovarian steroidogenesis and hepatic lipid metabolism in PCOS–NAFLD comorbidity. Additionally, GO and KEGG enrichment analyses indicated that FCGBP-associated genes were involved in inflammatory responses, innate immunity, and NOD-like receptor signaling, supporting its potential role as a regulatory hub at the intersection of immune, hormonal, and metabolic pathways. Although the precise signaling mechanisms remain to be elucidated, our findings provide a strong experimental foundation for positioning FCGBP as a cross-tissue regulatory factor in complex endocrine–metabolic disorders.

FCGBP may exert its effects through multiple potential pathways, including modulation of inflammatory signaling (e.g., NOD-like receptor or NF-κB pathways), indirect regulation of gene expression networks, or interaction with upstream regulatory factors rather than direct binding to target proteins (29–31). In addition, epigenetic mechanisms such as DNA methylation or transcriptional regulation cannot be excluded. However, the current evidence is based on expression and loss-of-function experiments and does not establish direct mechanistic links between FCGBP and steroidogenesis or lipid metabolism pathways. Further mechanistic studies are required to elucidate the detailed regulatory network of FCGBP.

Methodologically, we adopted a three-tiered strategy combining transcriptomic co-expression analysis, multi-algorithm feature selection, and *in vivo*/*in vitro* functional validation. By integrating LASSO regression, Random Forest, and XGBoost models, we improved feature selection reliability and mitigated potential overfitting through a nested cross-validation framework, enhancing the reliability and reproducibility of candidate gene identification. Compared to traditional single-factor analyses, our approach provides a more reliable framework for discovering shared molecular regulators across diseases. However, the previously observed perfect classification performance (AUC = 1.000) likely reflects overfitting under small-sample conditions. Nested cross-validation results provide a more realistic estimate of model performance. Moreover, calibration and nomogram analyses derived from the same dataset should be interpreted with caution. FCGBP, reproducibly identified across datasets and supported by multiple machine learning models, and validated through both cellular and animal experiments, highlights its potential as a promising target for further investigation. Importantly, the primary aim of this study was to explore the molecular role of FCGBP rather than to establish a fully validated animal model of PCOS–NAFLD comorbidity.

Our findings offer a molecular rationale for the development of diagnostic and therapeutic tools targeting PCOS–NAFLD comorbidity. FCGBP may serve as a potential biomarker for identifying shared molecular features between PCOS and NAFLD and may help predict the risk of hepatic complications in PCOS patients. Moreover, targeting FCGBP or its downstream signaling pathways could offer novel strategies for dual disease modulation. Nonetheless, certain limitations should be acknowledged. First, the relatively small sample size of the public datasets, particularly for PCOS (n=10), together with the lack of detailed clinical information (e.g., diagnostic criteria and comorbidities such as diabetes or hypertension), may introduce confounding bias and limit the generalizability, external validity, and clinical interpretability of the identified DEGs. In addition, the limited sample size may increase the risk of false positives and reduce statistical power. Although LOO resampling showed consistent detection of key genes with relatively stable effect sizes, their statistical significance was sensitive to multiple testing correction, indicating that these findings should be interpreted as exploratory rather than definitive. Second, the PCOS and NAFLD datasets were derived from independent cohorts rather than patients with confirmed comorbidity, and clinical phenotype data were not available, which limits direct assessment of FCGBP in comorbid populations. Third, although protein-level validation of FCGBP was performed *in vitro*, *in vivo* protein expression was not assessed due to limited tissue availability. In addition, the animal model was used to mimic key features of PCOS–NAFLD but was not comprehensively evaluated for all classical phenotypes, which may reduce the translational strength of our findings. Furthermore, HepG2 cells, a hepatocellular carcinoma-derived cell line, were used as an *in vitro* model and may not fully recapitulate normal hepatocyte physiology, thereby limiting physiological relevance. Finally, although both FCGBP and VAV3 were identified as candidate hub genes through machine learning, only FCGBP was functionally validated in this study. The lack of experimental validation for VAV3 may introduce potential selection bias, and its biological role in PCOS–NAFLD comorbidity warrants further investigation. Future studies based on well-characterized clinical cohorts with clearly defined diagnostic criteria and comprehensive clinical data are warranted to validate FCGBP expression in clinical samples and further elucidate its involvement in canonical signaling pathways such as NF-κB or JAK/STAT.

In summary, our study integrates transcriptomic analysis and machine learning to identify and validate FCGBP as a novel regulator of PCOS–NAFLD comorbidity. We propose FCGBP as a candidate gene associated with both ovarian hormonal imbalance and hepatic steatosis, providing preliminary insights into the molecular mechanisms underlying reproductive–metabolic disease intersections.

## 5 Conclusion

This study identified FCGBP as a key co-expressed gene in PCOS–NAFLD comorbidity using transcriptomic and machine learning approaches. Functional assays showed that FCGBP knockdown restored estrogen synthesis in ovarian cells and reduced lipid accumulation in hepatocytes by regulating CYP19A1, HSD17B1, SCD1, and ATGL. These findings suggest that FCGBP may be associated with both hormonal imbalance and hepatic steatosis. Targeting FCGBP may represent a potential dual-organ intervention strategy for PCOS–NAFLD comorbidity. However, further validation in well-characterized clinical cohorts and mechanistic studies is required to confirm its translational relevance.

## Data Availability

The original contributions presented in the study are included in the article/**Supplementary Material**. Further inquiries can be directed to the corresponding author.
