# Supplementary material for: FCGBP links hormonal imbalance and hepatic steatosis in PCOS–NAFLD comorbidity: an integrative bioinformatics and experimental study
Source: Front Endocrinol (Lausanne). 2026 May 18;17:1750425. doi: 10.3389/fendo.2026.1750425 (PMC13222972; doi:10.3389/fendo.2026.1750425)
Supplement: Supplementary file 1 [file DataSheet1.docx]

Table S1. Leave-one-out (LOO) stability analysis of core genes across individual iterations.

| **Gene** | **Run** | **Detected** | **Rank** | **logFC** |
| --- | --- | --- | --- | --- |
| FCGBP | LOO_1 | 1 | 105 | 2.4002 |
| FCGBP | LOO_2 | 1 | 18 | 2.6398 |
| FCGBP | LOO_3 | 1 | 97 | 1.7751 |
| FCGBP | LOO_4 | 1 | 70 | 2.3262 |
| FCGBP | LOO_5 | 1 | 96 | 2.2469 |
| FCGBP | LOO_6 | 1 | 15 | 2.4328 |
| FCGBP | LOO_7 | 1 | 69 | 2.2458 |
| FCGBP | LOO_8 | 1 | 59 | 2.1791 |
| FCGBP | LOO_9 | 1 | 61 | 2.2566 |
| FCGBP | LOO_10 | 1 | 63 | 2.2145 |
| VAV3 | LOO_1 | 1 | 439 | 1.1024 |
| VAV3 | LOO_2 | 1 | 85 | 1.4761 |
| VAV3 | LOO_3 | 1 | 143 | 1.3879 |
| VAV3 | LOO_4 | 1 | 146 | 1.3648 |
| VAV3 | LOO_5 | 1 | 232 | 1.3156 |
| VAV3 | LOO_6 | 1 | 88 | 1.3712 |
| VAV3 | LOO_7 | 1 | 186 | 1.2718 |
| VAV3 | LOO_8 | 1 | 176 | 1.2604 |
| VAV3 | LOO_9 | 1 | 134 | 1.3053 |
| VAV3 | LOO_10 | 1 | 96 | 1.3661 |

Table S2. Summary statistics of LOO reproducibility and effect size stability.

| **Gene** | **Reproducibility** | **Rank median** | **Rank range** | **logFC mean** | **logFC SD** |
| --- | --- | --- | --- | --- | --- |
| FCGBP | 10/10 | 66 | 15–105 | 2.2717 | 0.2214 |
| VAV3 | 10/10 | 144.5 | 85–439 | 1.3222 | 0.0995 |

Table S3. Sensitivity analysis of DEG identification under different thresholds.

| **logFC cutoff** | **FDR cutoff** | **DEG number** | **FCGBP is DEG** | **FCGBP rank** |
| --- | --- | --- | --- | --- |
| 0.3 | 0.10 | 4 | no | NA |
| 0.3 | 0.05 | 0 | no | NA |
| 0.585 | 0.10 | 4 | no | NA |
| 0.585 | 0.05 | 0 | no | NA |
| 1.0 | 0.10 | 4 | no | NA |
| 1.0 | 0.05 | 0 | no | NA |

Table S4. Nested cross-validation performance of machine learning models for PCOS classification.

| **Model** | **AUC** | **Bootstrap mean AUC** | **95% CI (lower)** | **95% CI (upper)** |
| --- | --- | --- | --- | --- |
| LASSO | 0.955 | 0.952 | 0.925 | 0.979 |
| Random Forest | 0.912 | 0.908 | 0.875 | 0.938 |
| XGBoost | 0.904 | 0.870 | 0.832 | 0.932 |
| Ensemble (mean) | 0.932 | 0.928 | 0.895 | 0.958 |

Table S5. Frequency of core gene selection across nested cross-validation iterations

| **Gene** | **LASSO (n=10)** | **Random Forest (n=10)** | **XGBoost (n=10)** |
| --- | --- | --- | --- |
| FCGBP | 5 | 6 | 4 |
| VAV3 | 8 | 5 | 3 |


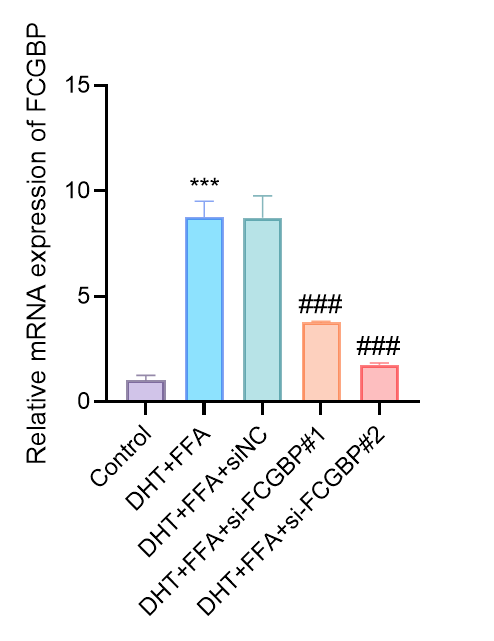


Figure S1. Validation of FCGBP knockdown efficiency using two independent siRNAs in HepG2 cells. Data are presented as mean ± SD (n = 3 independent experiments). ***P < 0.001 vs. Control; ###P < 0.001 vs. DHT+FFA+siNC.


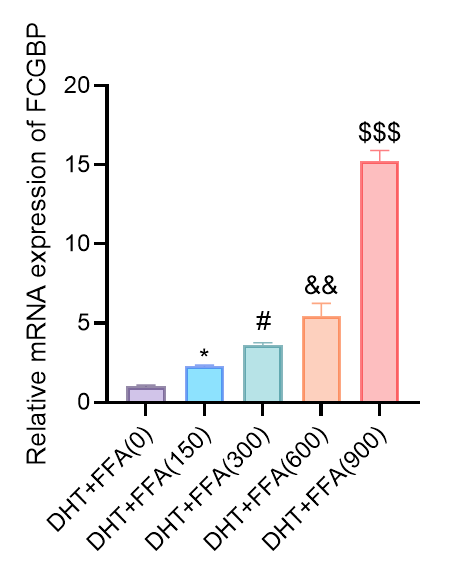


Figure S2. Dose-dependent induction of FCGBP expression by DHT in HepG2 cells. Data are presented as mean ± SD (n = 3 independent experiments). *P < 0.05 vs. DHT+FFA(0); #P < 0.05 vs. DHT+FFA(150); &&P < 0.01 vs. DHT+FFA(300); $$$P < 0.001 vs. DHT+FFA(600).
